# The Homœopathic Controversy

**Published:** 1890-01

**Authors:** 


### THE HOMŒOPATHIC CONTROVERSY.

From the *New York Sun*, January 8th.

There seems to be no important principle of medicine or of morals involved in the demand of the Homœopathic County Society for the reconstruction of the medical board of the Homœopathic Hospital on Ward's Island, New York. The Commissioners of Charities and Correction are asked to make the change simply on the ground that some of the physicians of the hospital do not belong to the society.

As we understand it, there is no practical difference between the methods of treatment pursued by members of the society and physicians of the homœopathic school who are outside of that association. According to Dr. Guernsey, the President of the Ward's Island Medical Board, not a single strict homœopathist now remains in New York. The two hundred members of the society and the two hundred other practitioners whom Commissioner Simmons speaks of as claiming the title of homœopathists, both use the remedies of the old school of medicine when they deem them needful. Therefore the society, in asking for the reorganization of the hospital staff, does not contemplate any change in the methods of treatment. It wants only that the principle shall be established by the Commissioners of making appointments solely from among the members of the society.

That is a purely private matter of no public concern. But it is of public interest and importance that the homœopathic school, in all its divisions, substantially acknowledges that it no longer adheres to the principles of Hahnemann to the exclusion of all other. As the County Society defines a homœopathic physician, he is simply a member of that association. As the resolution of the Ward's Island staff defines him, he is merely a physician who believes in the principle of like cures like, but because of it is not deterred from "recognizing and making use of the results of any experience," "or any therapeutic fact founded on experiments and verified by experience, so far as in his individual judgment they shall tend to promote the welfare of those under his professional care." That is the actual practice at the hospital, and it is not denied that it is the actual practice among homœopathic physicians generally at this period.

The line of demarcation between the homœopathic and regular practitioner is therefore almost entirely obliterated. The principle of like cures like and of minimum doses is, of course, abandoned, when it is no longer made of universal application; and the theory that the medical art should make use of whatever experiment and experience have proved efficacious for the alleviation of disease, lies at the basis of the old practice. The regular school is not debarred from using homœopathic remedies, water-cure remedies, or any other methods of treatment, so long as they serve its purpose. It has the whole world to choose from, and the homœopathists are exercising the same liberty. Hence homœopathic physicians have joined the County Society of the regular school as brethren in science; and the abolition of the old rule of the regulars forbidding consultation with homœopaths is tending still further to wipe away the distinctions between the two. There are also many homœopaths who would give up that title as no longer describing their school in its later development. They would call themselves the New School, and yet there does not seem to be a logical reason for any separation at all in the ranks of medicine if on all sides the principle announced by the Ward's Island staff is adopted and followed.

It is fortunate for the healing art that such substantial union is the plain drift of the period. That art is old, and yet its greatest progress has been made within very recent years; and along lines now pursued its future advance is sure to be even more rapid. But this progress requires that investigation and experiment shall not be hampered by theory. There should only be one school of medicine, the school which addresses itself to making a science of the art.

[NOTE.—We specially commend the above extract to the attention of our readers.

It gives a good idea of the humiliating spectacle the homœopathic school presents before the world by reason of the atrocious conduct and teachings of men who only *pretend* to practice Homœopathy; but who really despise the system as much as the most hostile practitioner of the old school.—EDS.]

